# P-131. Study of Enteric Parasitic Infections to Delineate Low Grade Inflammation in Patients with Functional Dyspepsia

**DOI:** 10.1093/ofid/ofae631.336

**Published:** 2025-01-29

**Authors:** Sanjana Pant, Bijay Ranjan Mirdha, Govind Makharia, Prasenjit Das, Ranveer Singh Jadon

**Affiliations:** All India Institute of Medical Sciences, Delhi, Delhi, Delhi, India; All India Institute of Medical Sciences, Delhi, Delhi, Delhi, India; All India Institute of Medical Sciences, Delhi, Delhi, Delhi, India; All India Institute of Medical Sciences, Delhi, Delhi, Delhi, India; All India Institute of Medical Sciences, Delhi, Delhi, Delhi, India

## Abstract

**Background:**

Growing evidence of chronic low-grade intestinal inflammation in post-infectious functional gastrointestinal disorders (FGID), including functional dyspepsia (FD), have been observed. Present study aimed to investigate the role of enteric parasitic infestations along with *Helicobacter pylori* and associated inflammatory cellular components among FD patients.

**Figure:**
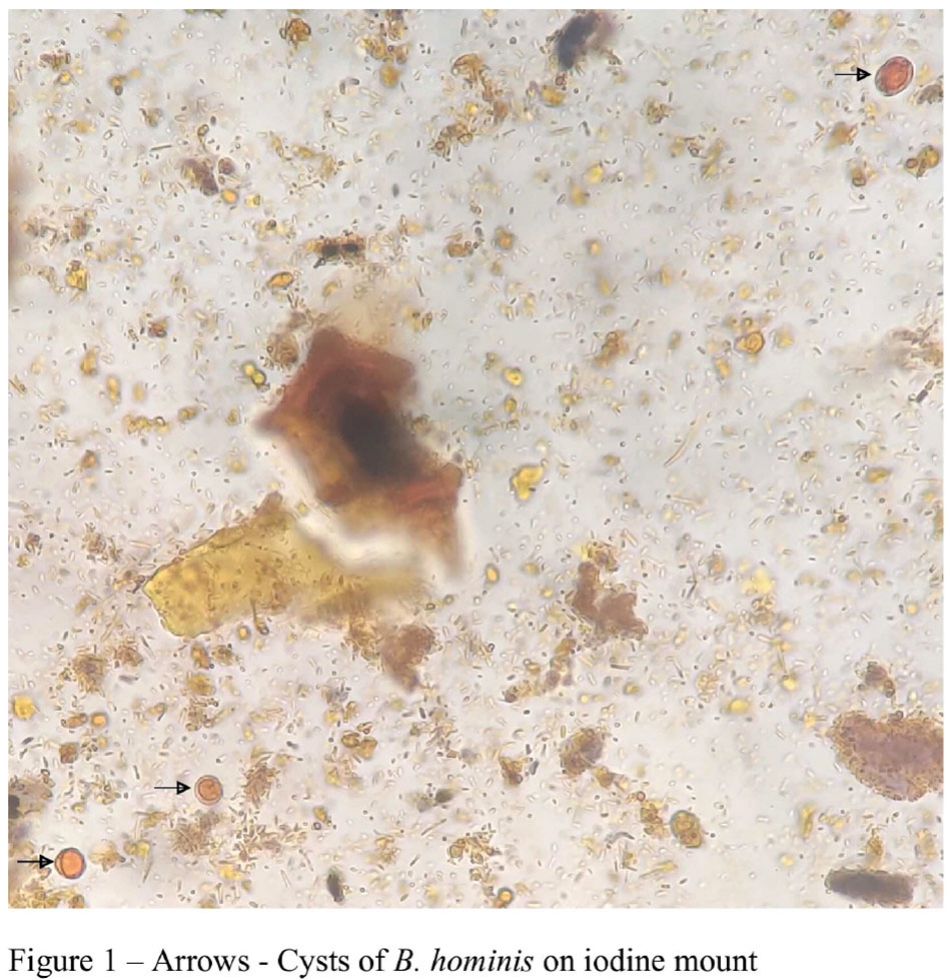

**Methods:**

Prospective observational study included 183 adult patients with symptoms of FD consistent with the ROME IV criteria. All these patients were subjected to clinical evaluation, routine blood tests, and stool sample analyses for parasitological examination. Upper gastrointestinal endoscopic biopsy samples were examined for the presence of *H. pylori* and to extrapolate cellular components of low-grade inflammation.

**Figure:**
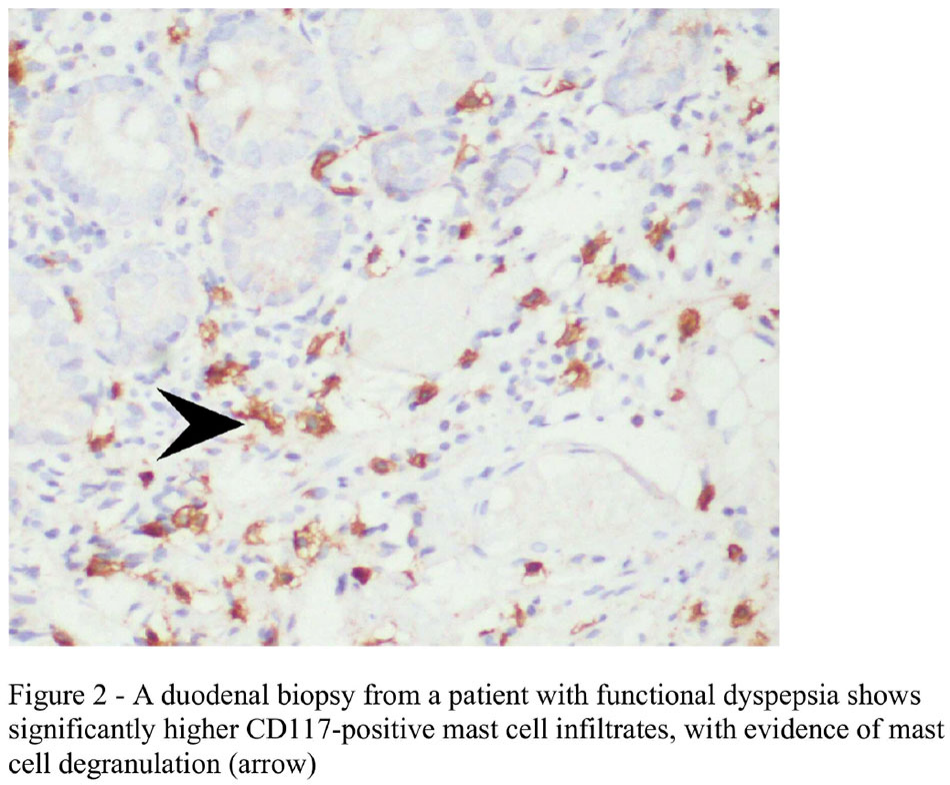

**Results:**

Among the 183 individuals, (85/183) 46.4% and (98/183) 53.6% belonged to the subtype post-prandial distress syndrome (PPDS) and epigastric pain syndrome (EPS) respectively. 46/183 (25.14%) of the stool samples tested positive for parasites, most common being *Blastocystis* species. The distribution of *Blastocytis* cells were 14/85 (16.47%) for PPDS and 16 (16.32%) for EPS patients. Histopathological analysis revealed significantly elevated duodenal mast cells and their degranulation among 85.24% and 76.5% FD patients respectively whereas, duodenal eosinophilia, duodenal eosinophil degranulation, and duodenal intra-epithelial lymphocytosis were observed in 3.28%, 15.3%, 1.7% respectively. Patients of the EPS subtype with intestinal parasitosis had elevated duodenal mast cell count, albeit statistically non-significant (*p* value = 0.078). There was no significant correlation between intestinal parasitosis or *H. pylori* with any of the clinical parameters examined, other than intestinal parasitosis with anemia (PPDS, *p*-value < 0.02).

**Figure:**
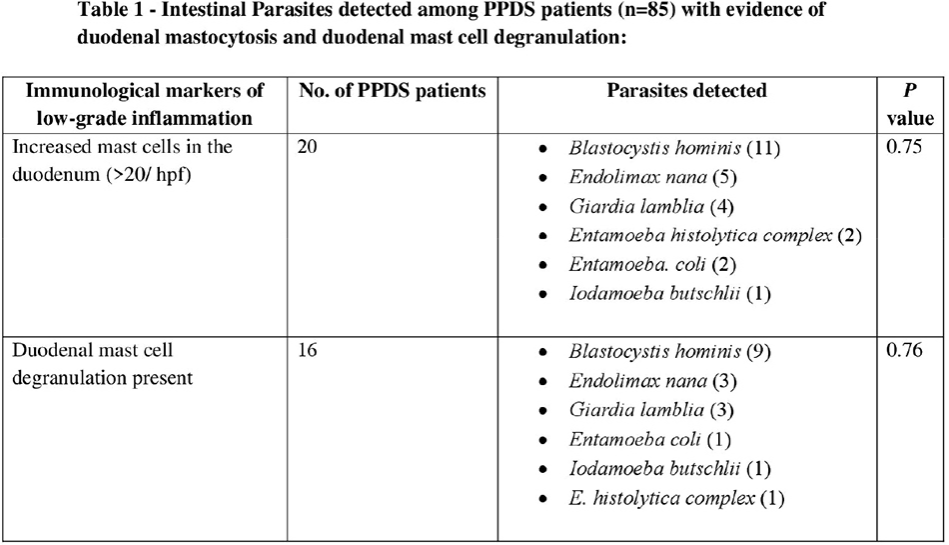

**Conclusion:**

Presence of duodenal mast cells along with the presence of *Blastocystis* cells in some of the FD patients suggests their potential involvement in symptom generation, albeit lack of experimental studies. Our findings also give impetus to further understand the complex interplay between elevated duodenal mast cell count and mast cell degranulation with FD symptoms.

**Figure:**
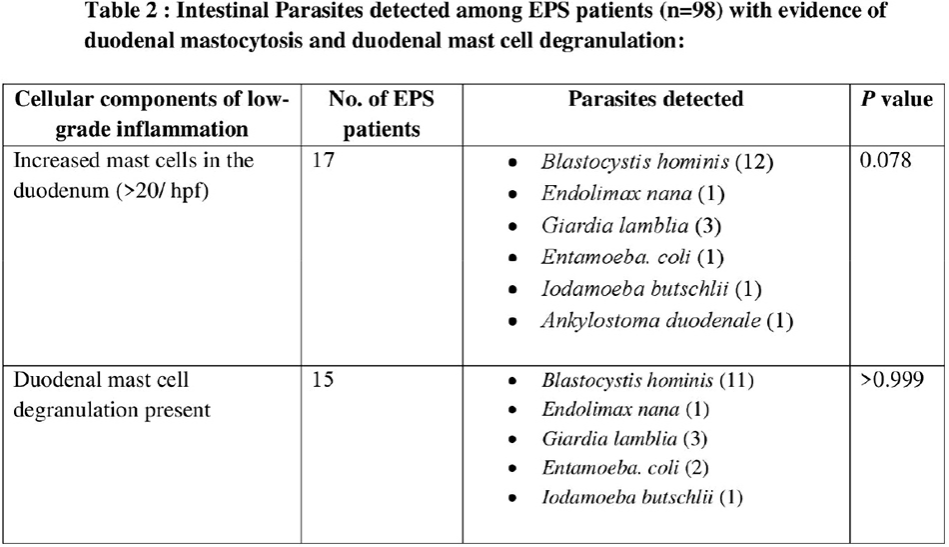

**Disclosures:**

**All Authors**: No reported disclosures

